# Supplementary material for: Colombia’s bioregions as a source of useful plants
Source: PLoS One. 2021 Aug 27;16(8):e0256457. doi: 10.1371/journal.pone.0256457 (PMC8396733; doi:10.1371/journal.pone.0256457)
Supplement: S1 File — (DOCX) [file pone.0256457.s001.docx]

**S1 File**

**Colombia’s Flora: Data processing flow and results.**

**Script sections:**

**1. Standardize and join data of plants of Colombia (SiB Colombia and GBIF - Global Biodiversity Information Facility)**

**2. Match names against POWO taxonomy using PyKew**

**3. Geographic cleaning using CoordinateCleaner**

**4. Results and Summary**

**
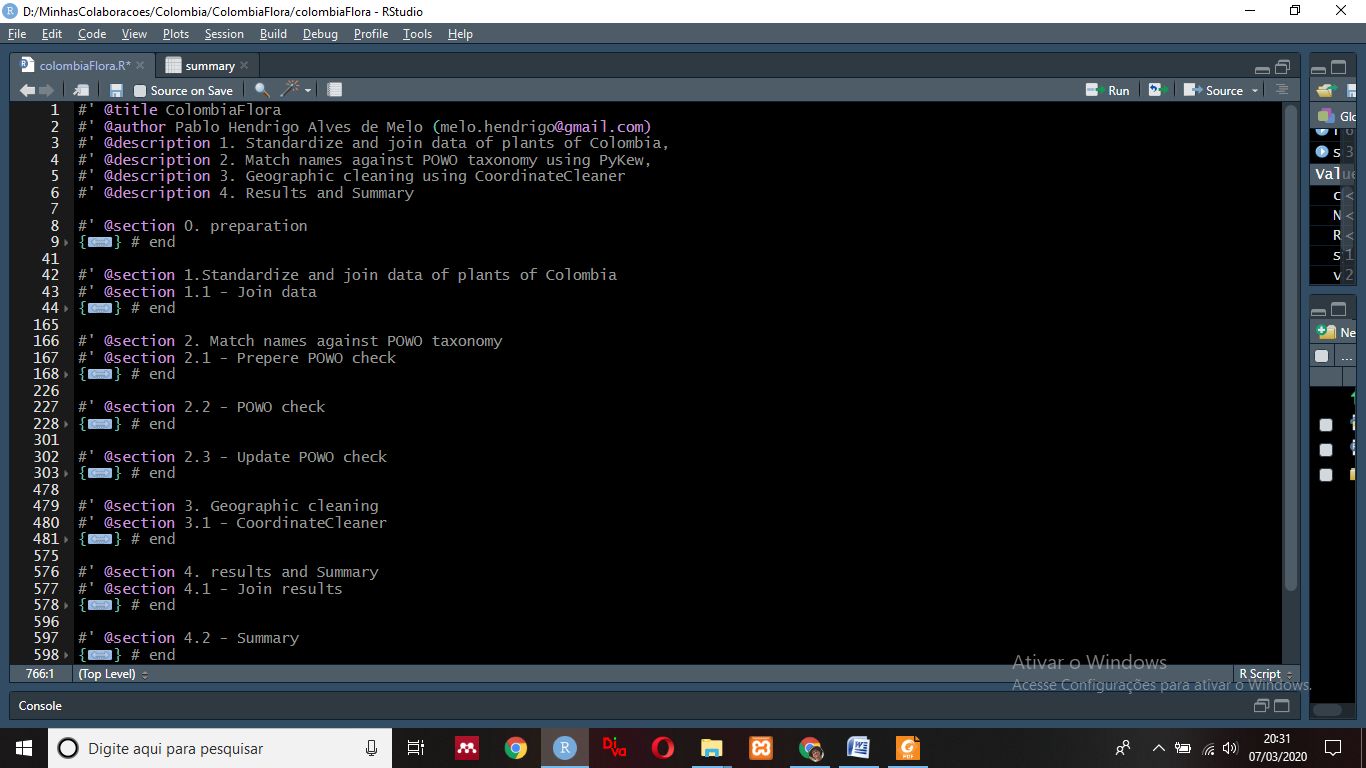
**

**Merge columns:**

**source (GBIF or SIB)**

**scientificName_Source**

**taxonRank_Source**

**occurrenceID**

**basisOfRecord**

**modified**

**institutionCode**

**collectionCode**

**catalogNumber**

**identificationQualifier**

**identifiedBy**

**dateIdentified**

**typeStatus**

**recordNumber**

**recordedBy**

**countryCode**

**stateProvince**

**municipality**

**locality**

**year**

**month**

**day**

**decimalLatitude**

**decimalLongitude**

**elevation**

**occurrenceRemarks**

**fieldNotes**

**vernacularName**

**samplingProtocol**

**Results columns**

**Match names against POWO taxonomy:**

**.submittedToPOWO**

**search_unique_POWO (species name without author used in POWO search)**

**nomenclaturestatus_POWO**

**author_POWO**

**kingdom_POWO**

**group_POWO**

**order_POWO**

**family_POWO**

**name_POWO**

**rank_POWO**

**url_POWO**

**fqId_POWO**

**synonyms_homonyms_POWO**

**Geographic cleaning:**

**.submittedToCoordinateCleaner**

**.val (Invalid lat/lon Coordinates)**

**.equ (Records with Identical lat/lon)**

**.zer (Zero Coordinates)**

**.cap (Coordinates in Vicinity of Country Capitals)**

**.cen (Coordinates in Vicinity of Country and Province Centroids)**

**.sea (Non-terrestrial Coordinates)**

**.urb (Records Inside Urban Areas)**

**.con (Coordinates Outside their Reported Country)**

**.inst (Records in the Vicinity of Biodiversity Institutions)**

**.dpl (Duplicated Records)**

**.summary**

**Summary results**

**S1 Table. Summary.**

| Description | Value |
| --- | --- |
| total of records | 4020480 |
| total of records GBIF | 2130122 |
| total of records SIB | 1890358 |
| number of records submitted to POWO | 3273381 |
| number of records not submitted to POWO | 747099 |
| unique species names submitted to POWO | 53656 |
| unique names (form, var., subsp. and species) returned to POWO | 34276 |
| nomenclature status POWO (n.rec.): accepted | 2675608 |
| nomenclature status POWO (n.rec.): no results | 268131 |
| nomenclature status POWO (n.rec.): synonym | 379956 |
| nomenclature status POWO (n.rec.): synonym without accepted name resolution | 845 |
| nomenclature status POWO (n.rec.): NA | 695940 |
| nomenclature status POWO (n.rec.): synonyms homonyms POWO | 34276 |
| group (n.rec.): Angiosperms | 2849240 |
| group (n.rec.): Gymnosperms | 5009 |
| group (n.rec.): is not vascular plant | 6388 |
| group (n.rec.): Lycophytes and ferns | 194927 |
| group (n.rec.): NA | 964916 |
| taxon rank POWO (n.rec.): Form | 213 |
| taxon rank POWO (n.rec.): Genus | 6 |
| taxon rank POWO (n.rec.): Species | 3041658 |
| taxon rank POWO (n.rec.): Subspecies | 10377 |
| taxon rank POWO (n.rec.): Variety | 3310 |
| taxon rank POWO (n.rec.): NA | 964916 |
| number of records submitted to CoordinateCleaner | 3132572 |
| number of records not submitted to CoordinateCleaner | 887908 |
| Geographic cleaning: Invalid lat/lon Coordinates | 0 |
| Geographic cleaning: Records with Identical lat/lon | 3597 |
| Geographic cleaning: Zero Coordinates | 3886 |
| Geographic cleaning: Coordinates in Vicinity of Country Capitals | 37208 |
| Geographic cleaning: Coordinates in Vicinity of Country and Province Centroids | 16671 |
| Geographic cleaning: Non-terrestrial Coordinates | 37239 |
| Geographic cleaning: Records Inside Urban Areas | 56113 |
| Geographic cleaning: Coordinates Outside their Reported Country | 37878 |
| Geographic cleaning: Records in the Vicinity of Biodiversity Institutions | 6370 |
| Geographic cleaning: Duplicated Records | 2963613 |
