# Supplementary material for: Colombia’s bioregions as a source of useful plants
Source: PLoS One. 2021 Aug 27;16(8):e0256457. doi: 10.1371/journal.pone.0256457 (PMC8396733; doi:10.1371/journal.pone.0256457)
Supplement: S2 File — (DOCX) [file pone.0256457.s002.docx]

**S2 File**

**S2.1 Table. Dataset of vascular plants and a subset of useful plants of Colombia.**

| **Characteristics** | **All vascular plants** | **Useful plants** | |
| --- | --- | --- | --- |
|  |  | **Number** | **Per cent of all vascular plants** |
| **Unique locality records number (i.e. unique combinations of species x localities)** | 522257 | 197166 | 37.75 |
| **Unique species number (POWO accepted)** | 23961 | 3870 | 16.15 |
| **Unique families number (POWO accepted)** | 281 | 228 | 81.14 |
| **Average number of records per species** | 21.79 | 50.95 | NA |
| **Point endemics number (i.e. known from a single record or locality)** | 4721 | 221 | 4.68 |
| **Most abundant species (number of unique records)** | *Gaultheria*  *myrsinoides*  (1015) | *Quercus humboldtii*  (877) | NA |

**S2.2 Table. Generalized ecosystem types (bioregions).**

| **Olson’s name** | **Name in this study** | **Regions** | **Analysis units for this study (bioregions)** |
| --- | --- | --- | --- |
| Desert and xeric shrublands | Desert and xeric shrublands | Caribbean | Desert and xeric shrublands |
| Mangroves | Mangroves | Caribbean and Pacific | Mangroves |
| Montane grasslands and shrublands | Paramos | Andes | Andean paramos |
| Montane grasslands and shrublands | Paramos | Caribbean | Caribbean paramos |
| Tropical and subtropical dry broadleaf forests | Dry forests | Caribbean | Caribbean dry forests |
| Tropical and subtropical dry broadleaf forests | Dry forests | Andes | Andean dry forests |
| Tropical and subtropical dry broadleaf forests | Dry forests | Llanos | Llanos dry forests |
| Tropical and subtropical grasslands, savannas and shrublands | Savannas | Llanos | Savannas |
| Tropical and subtropical moist broadleaf forests | Moist forests | Caribbean | Caribbean moist forests |
| Tropical and subtropical moist broadleaf forests | Moist forests | Pacific | Pacific moist forests |
| Tropical and subtropical moist broadleaf forests | Moist forests | Andes | Andean moist forests |
| Tropical and subtropical moist broadleaf forests | Moist forests | Llanos | Llanos moist forests |
| Tropical and subtropical moist broadleaf forests | Moist forests | Amazon | Amazon moist forests |

**S2.3 Table. Transformed areas in bioregions.**

| **ID** | **Bioregions** | **Extent measured as a number of 10x10 km grid cells** | | **Relative size of transformed areas, per cent of (4)** |
| --- | --- | --- | --- | --- |
|  |  | **Transformed areas excluded** | **Transformed areas included** |  |
| 1 | 2 | 3 | 4 | 5 |
| **4** | **Paramos - Caribe** | 17 | 17 | 0.00 |
| **12** | **Moist forest - Llanos** | 1374 | 1466 | 6.28 |
| **9** | **Moist forest - Amazonas** | 3162 | 3442 | 8.13 |
| **8** | **Savannas - Llanos** | 1535 | 1757 | 12.64 |
| **3** | **Paramos - Andes** | 133 | 160 | 16.88 |
| **13** | **Moist forest - Pacifico** | 559 | 679 | 17.67 |
| **2** | **Mangroves** | 77 | 97 | 20.62 |
| **1** | **Deserts and xeric shrublands - Caribe** | 201 | 349 | 42.41 |
| **10** | **Moist forest - Andes** | 1521 | 2952 | 48.48 |
| **6** | **Dry forest - Llanos** | 134 | 291 | 53.95 |
| **11** | **Moist forest - Caribe** | 441 | 1059 | 58.36 |
| **5** | **Dry forest - Andes** | 95 | 327 | 70.95 |
| **7** | **Dry forest - Caribe** | 77 | 314 | 75.48 |
| **14** | **Transformed areas** | 3584 | NA | NA |

**S2.4 Table. All vascular plants and useful plants of Colombia: species and collection localities in bioregions (10x10 km grid cells).** Record numbers don’t add up to the totals in S2.1 Table, because not all collection localities obtained Bioregion value.

| **ID** | **Bioregion** | **Number of 10x10 km grid cells** | **All vascular plants** | | **Useful plants** | |
| --- | --- | --- | --- | --- | --- | --- |
|  |  |  | **Records** | **Species** | **Records** | **Species** |
| **14** | **Transformed areas** | 3584 | 202598 | 17056 | 85614 | 3497 |
| **10** | **Moist forests - Andes** | 1521 | 136021 | 13909 | 42258 | 2781 |
| **9** | **Moist forests - Amazonas** | 3162 | 61036 | 6650 | 21428 | 1612 |
| **13** | **Moist forests - Pacifico** | 559 | 27332 | 5168 | 9336 | 1336 |
| **12** | **Moist forests - Llanos** | 1374 | 23677 | 3953 | 9964 | 1279 |
| **11** | **Moist forests - Caribe** | 441 | 8702 | 3055 | 4216 | 1166 |
| **8** | **Savannas - Llanos** | 1535 | 18580 | 2938 | 8903 | 1151 |
| **3** | **Paramos - Andes** | 133 | 26890 | 3955 | 6265 | 816 |
| **5** | **Dry forests - Andes** | 95 | 3849 | 1470 | 2448 | 753 |
| **6** | **Dry forests - Llanos** | 134 | 3070 | 1300 | 1662 | 616 |
| **1** | **Deserts and xeric shrublands - Caribe** | 201 | 2290 | 1014 | 1301 | 556 |
| **7** | **Dry forests - Caribe** | 77 | 1658 | 922 | 753 | 394 |
| **2** | **Mangroves** | 77 | 1334 | 871 | 629 | 364 |
| **4** | **Paramos - Caribe** | 17 | 289 | 182 | 75 | 49 |

**S2.5 Table. Mean species richness across bioregions (all plants).**

| **ID** | **Bioregion** | **Study area** | | **Surveyed area** | | **Well surveyed area** | |
| --- | --- | --- | --- | --- | --- | --- | --- |
|  |  | **Mean richness** | **Area** | **Mean richness** | **Area** | **Mean richness** | **Area** |
| **0** | **NA** | 0.26 | 14585 | 23.83 | 157 | 71.00 | 44 |
| **1** | **Deserts and xeric shrublands - Caribe** | 9.23 | 201 | 20.38 | 91 | 60.35 | 26 |
| **2** | **Mangroves** | 14.29 | 77 | 34.38 | 32 | 63.53 | 15 |
| **3** | **Paramos - Andes** | 94.42 | 133 | 104.65 | 120 | 147.77 | 83 |
| **4** | **Paramos - Caribe** | 13.24 | 17 | 13.24 | 17 | 39.00 | 4 |
| **5** | **Dry forests - Andes** | 30.78 | 95 | 34.40 | 85 | 60.05 | 44 |
| **6** | **Dry forests - Llanos** | 14.80 | 134 | 28.74 | 69 | 82.62 | 21 |
| **7** | **Dry forests - Caribe** | 14.74 | 77 | 27.02 | 42 | 82.92 | 12 |
| **8** | **Savannas - Llanos** | 6.74 | 1535 | 20.94 | 494 | 74.71 | 112 |
| **9** | **Moist forests - Amazonas** | 13.44 | 3162 | 44.59 | 953 | 118.98 | 329 |
| **10** | **Moist forests - Andes** | 52.47 | 1521 | 72.55 | 1100 | 134.21 | 570 |
| **11** | **Moist forests - Caribe** | 15.02 | 441 | 29.18 | 227 | 75.28 | 78 |
| **12** | **Moist forests - Llanos** | 9.19 | 1374 | 34.79 | 363 | 90.20 | 127 |
| **13** | **Moist forests - Pacifico** | 34.96 | 559 | 61.45 | 318 | 131.90 | 139 |
| **14** | **Transformed areas** | 37.78 | 3584 | 51.29 | 2640 | 104.69 | 1202 |

**S2.6 Table. Mean species richness across bioregions (useful plants).**

| **ID** | **Bioregion** | **Study area** | | **Surveyed area** | | **Well surveyed area** | |
| --- | --- | --- | --- | --- | --- | --- | --- |
|  |  | **Mean richness** | **Area** | **Mean richness** | **Area** | **Mean richness** | **Area** |
| **0** | **NA** | 0.12 | 14585 | 13.01 | 138 | 50.79 | 24 |
| **1** | **Deserts and xeric shrublands - Caribe** | 5.38 | 201 | 13.68 | 79 | 48.19 | 16 |
| **2** | **Mangroves** | 6.52 | 77 | 15.69 | 32 | 40.14 | 7 |
| **3** | **Paramos - Andes** | 22.47 | 133 | 28.47 | 105 | 48.65 | 55 |
| **4** | **Paramos - Caribe** | 3.53 | 17 | 5.00 | 12 | 19.00 | 1 |
| **5** | **Dry forests - Andes** | 19.28 | 95 | 22.07 | 83 | 43.88 | 34 |
| **6** | **Dry forests - Llanos** | 7.92 | 134 | 16.58 | 64 | 57.27 | 15 |
| **7** | **Dry forests - Caribe** | 6.83 | 77 | 13.49 | 39 | 52.00 | 7 |
| **8** | **Savannas - Llanos** | 3.19 | 1535 | 12.10 | 404 | 50.08 | 66 |
| **9** | **Moist forests - Amazonas** | 4.78 | 3162 | 19.78 | 764 | 67.30 | 176 |
| **10** | **Moist forests - Andes** | 16.39 | 1521 | 25.70 | 970 | 61.51 | 343 |
| **11** | **Moist forests - Caribe** | 7.28 | 441 | 16.38 | 196 | 53.11 | 44 |
| **12** | **Moist forests - Llanos** | 3.84 | 1374 | 17.89 | 295 | 51.33 | 84 |
| **13** | **Moist forests - Pacifico** | 12.15 | 559 | 24.79 | 274 | 65.42 | 86 |
| **14** | **Transformed areas** | 16.23 | 3584 | 23.91 | 2433 | 59.43 | 803 |

**S2.7 Table. Plant uses across bioregions represented by unique collection localities.** Bioregion names as in S2.8 Table.

| **Bioregion ID** | **Animal Food** | **Environmental Uses** | **Fuels** | **Gene Sources** | **Human Food** | **Invertebrate Food** | **Materials** | **Medicines** | **Poisons** | **Social Uses** |
| --- | --- | --- | --- | --- | --- | --- | --- | --- | --- | --- |
| **1** | 352 | 470 | 205 | 242 | 448 | 137 | 701 | 1109 | 273 | 137 |
| **2** | 183 | 241 | 95 | 105 | 280 | 23 | 365 | 510 | 97 | 151 |
| **3** | 652 | 1338 | 310 | 411 | 1230 | 216 | 1248 | 5041 | 385 | 466 |
| **4** | 14 | 21 | 11 | 7 | 25 | 5 | 25 | 60 | 7 | 13 |
| **5** | 763 | 1073 | 422 | 520 | 1006 | 329 | 1456 | 2186 | 541 | 489 |
| **6** | 256 | 399 | 89 | 165 | 344 | 50 | 657 | 1367 | 132 | 159 |
| **7** | 201 | 228 | 94 | 152 | 249 | 53 | 384 | 635 | 137 | 132 |
| **8** | 1445 | 1668 | 622 | 644 | 1761 | 382 | 4187 | 7111 | 785 | 747 |
| **9** | 1910 | 2850 | 1069 | 1138 | 4257 | 378 | 9923 | 16184 | 1061 | 1911 |
| **10** | 5908 | 11575 | 3130 | 4592 | 10250 | 2004 | 16315 | 33649 | 4390 | 4097 |
| **11** | 724 | 1093 | 482 | 556 | 1215 | 260 | 2080 | 3360 | 567 | 430 |
| **12** | 1200 | 1802 | 723 | 631 | 2227 | 250 | 4637 | 7611 | 646 | 865 |
| **13** | 1374 | 2273 | 762 | 809 | 2782 | 324 | 4344 | 7428 | 972 | 1137 |
| **14** | 17786 | 30627 | 8574 | 15058 | 27507 | 6211 | 38591 | 71235 | 14253 | 10920 |

**S2.8 Table. Number of useful species per use category and botanical family.**

| **Family**  **(POWO)** | **Animal Food** | **Environmental Uses** | **Fuels** | **Gene Sources** | **Human Food** | **Invertebrate Food** | **Materials** | **Medicines** | **Poisons** | **Social Uses** |
| --- | --- | --- | --- | --- | --- | --- | --- | --- | --- | --- |
| Acanthaceae | 543 | 547 | 53 | 259 | 289 | 0 | 285 | 1399 | 230 | 0 |
| Achariaceae | 0 | 0 | 0 | 0 | 164 | 0 | 117 | 449 | 0 | 0 |
| Achatocarpaceae | 120 | 0 | 0 | 0 | 0 | 0 | 0 | 0 | 0 | 0 |
| Actinidiaceae | 0 | 0 | 0 | 0 | 3 | 0 | 0 | 288 | 0 | 0 |
| Aizoaceae | 31 | 31 | 0 | 31 | 31 | 0 | 0 | 31 | 13 | 0 |
| Alismataceae | 74 | 176 | 0 | 74 | 148 | 0 | 0 | 138 | 0 | 0 |
| Alstroemeriaceae | 0 | 7 | 0 | 7 | 7 | 0 | 0 | 264 | 0 | 0 |
| Altingiaceae | 0 | 1 | 0 | 0 | 1 | 0 | 1 | 1 | 0 | 0 |
| Amaranthaceae | 818 | 1050 | 16 | 1389 | 1495 | 0 | 1096 | 1972 | 385 | 1141 |
| Amaryllidaceae | 43 | 89 | 0 | 62 | 48 | 0 | 47 | 92 | 37 | 14 |
| Anacardiaceae | 654 | 968 | 603 | 663 | 980 | 690 | 2048 | 2432 | 823 | 595 |
| Annonaceae | 203 | 305 | 178 | 255 | 894 | 158 | 683 | 1960 | 226 | 178 |
| Apiaceae | 278 | 165 | 0 | 212 | 341 | 0 | 322 | 782 | 210 | 40 |
| Apocynaceae | 423 | 720 | 134 | 90 | 622 | 237 | 1646 | 3417 | 663 | 157 |
| Aquifoliaceae | 0 | 0 | 0 | 90 | 24 | 0 | 37 | 24 | 0 | 0 |
| Araceae | 138 | 1200 | 72 | 459 | 312 | 0 | 310 | 2453 | 372 | 46 |
| Araliaceae | 6 | 136 | 6 | 0 | 6 | 0 | 530 | 800 | 2 | 2 |
| Araucariaceae | 0 | 14 | 0 | 0 | 14 | 0 | 14 | 14 | 0 | 0 |
| Arecaceae | 4593 | 3608 | 2727 | 571 | 6588 | 30 | 7900 | 5331 | 31 | 4042 |
| Aristolochiaceae | 0 | 75 | 0 | 0 | 0 | 0 | 0 | 380 | 0 | 0 |
| Asparagaceae | 29 | 103 | 23 | 49 | 75 | 35 | 85 | 130 | 34 | 43 |
| Asphodelaceae | 0 | 18 | 0 | 4 | 4 | 0 | 7 | 19 | 0 | 0 |
| Aspleniaceae | 0 | 217 | 0 | 52 | 55 | 0 | 37 | 1243 | 34 | 0 |
| Asteraceae | 1298 | 1846 | 285 | 1150 | 1938 | 723 | 2183 | 9302 | 970 | 842 |
| Balanophoraceae | 0 | 0 | 0 | 0 | 0 | 0 | 0 | 101 | 0 | 0 |
| Balsaminaceae | 0 | 125 | 0 | 46 | 46 | 0 | 46 | 125 | 46 | 0 |
| Basellaceae | 3 | 12 | 0 | 3 | 16 | 0 | 3 | 17 | 0 | 3 |
| Bataceae | 0 | 0 | 0 | 0 | 0 | 0 | 20 | 20 | 0 | 0 |
| Begoniaceae | 0 | 50 | 0 | 50 | 32 | 0 | 0 | 327 | 0 | 0 |
| Berberidaceae | 0 | 0 | 0 | 0 | 0 | 0 | 0 | 239 | 0 | 0 |
| Betulaceae | 256 | 256 | 256 | 0 | 0 | 0 | 256 | 256 | 0 | 0 |
| Bignoniaceae | 254 | 788 | 466 | 190 | 386 | 310 | 1720 | 1779 | 219 | 240 |
| Bixaceae | 230 | 353 | 230 | 70 | 230 | 0 | 460 | 530 | 230 | 230 |
| Bonnetiaceae | 0 | 0 | 0 | 0 | 0 | 0 | 0 | 27 | 0 | 0 |
| Boraginaceae | 412 | 370 | 0 | 41 | 453 | 403 | 1055 | 1847 | 353 | 184 |
| Brassicaceae | 106 | 151 | 58 | 246 | 211 | 0 | 100 | 331 | 137 | 8 |
| Bromeliaceae | 0 | 493 | 0 | 49 | 114 | 0 | 273 | 243 | 0 | 0 |
| Brunelliaceae | 0 | 0 | 0 | 0 | 0 | 0 | 0 | 151 | 0 | 0 |
| Burseraceae | 220 | 220 | 220 | 0 | 65 | 220 | 979 | 1200 | 220 | 0 |
| Buxaceae | 0 | 1 | 0 | 0 | 0 | 0 | 1 | 1 | 0 | 0 |
| Cabombaceae | 0 | 1 | 0 | 0 | 0 | 0 | 0 | 1 | 0 | 0 |
| Cactaceae | 14 | 170 | 14 | 10 | 253 | 0 | 33 | 261 | 0 | 11 |
| Calceolariaceae | 0 | 98 | 0 | 0 | 0 | 0 | 0 | 50 | 0 | 0 |
| Calophyllaceae | 29 | 29 | 310 | 19 | 29 | 10 | 455 | 484 | 29 | 29 |
| Campanulaceae | 0 | 7 | 0 | 0 | 0 | 0 | 0 | 473 | 123 | 0 |
| Cannabaceae | 20 | 0 | 20 | 0 | 20 | 0 | 143 | 143 | 20 | 20 |
| Cannaceae | 86 | 88 | 0 | 2 | 88 | 0 | 2 | 4 | 0 | 0 |
| Capparaceae | 0 | 0 | 0 | 0 | 0 | 0 | 87 | 216 | 0 | 0 |
| Caprifoliaceae | 0 | 9 | 0 | 0 | 8 | 1 | 23 | 356 | 0 | 0 |
| Caricaceae | 79 | 112 | 0 | 265 | 112 | 79 | 79 | 174 | 79 | 79 |
| Caryocaraceae | 0 | 0 | 173 | 0 | 59 | 0 | 216 | 248 | 0 | 0 |
| Caryophyllaceae | 160 | 150 | 0 | 132 | 143 | 0 | 132 | 473 | 15 | 103 |
| Casuarinaceae | 12 | 12 | 12 | 0 | 12 | 0 | 12 | 12 | 0 | 12 |
| Celastraceae | 0 | 2 | 0 | 0 | 0 | 0 | 37 | 167 | 4 | 0 |
| Ceratophyllaceae | 0 | 9 | 0 | 0 | 0 | 0 | 0 | 9 | 0 | 0 |
| Chloranthaceae | 0 | 0 | 0 | 0 | 0 | 0 | 9 | 0 | 0 | 0 |
| Chrysobalanaceae | 90 | 92 | 90 | 90 | 189 | 90 | 781 | 480 | 0 | 26 |
| Cleomaceae | 0 | 0 | 0 | 0 | 0 | 0 | 0 | 36 | 0 | 0 |
| Clusiaceae | 0 | 165 | 129 | 0 | 289 | 0 | 622 | 2021 | 0 | 0 |
| Combretaceae | 109 | 152 | 150 | 109 | 111 | 62 | 389 | 339 | 64 | 62 |
| Commelinaceae | 149 | 146 | 0 | 0 | 149 | 0 | 92 | 626 | 0 | 149 |
| Connaraceae | 0 | 0 | 0 | 0 | 0 | 0 | 91 | 102 | 0 | 0 |
| Convolvulaceae | 521 | 751 | 15 | 593 | 540 | 105 | 608 | 1061 | 566 | 530 |
| Coriariaceae | 0 | 0 | 0 | 0 | 0 | 0 | 90 | 90 | 90 | 90 |
| Costaceae | 0 | 0 | 0 | 0 | 0 | 0 | 0 | 684 | 0 | 1 |
| Crassulaceae | 29 | 42 | 0 | 0 | 0 | 0 | 0 | 45 | 30 | 29 |
| Cucurbitaceae | 250 | 436 | 93 | 509 | 609 | 95 | 426 | 1035 | 375 | 100 |
| Cunoniaceae | 0 | 0 | 0 | 0 | 0 | 0 | 0 | 903 | 0 | 0 |
| Cupressaceae | 0 | 19 | 8 | 0 | 7 | 0 | 19 | 19 | 8 | 0 |
| Cyatheaceae | 0 | 0 | 0 | 0 | 0 | 0 | 0 | 495 | 0 | 0 |
| Cycadaceae | 0 | 12 | 0 | 12 | 12 | 1 | 12 | 12 | 1 | 1 |
| Cyclanthaceae | 0 | 421 | 0 | 141 | 141 | 0 | 141 | 149 | 0 | 0 |
| Cyperaceae | 403 | 484 | 84 | 34 | 187 | 112 | 724 | 1489 | 48 | 108 |
| Cyrillaceae | 0 | 32 | 0 | 0 | 0 | 32 | 32 | 0 | 0 | 0 |
| Dennstaedtiaceae | 0 | 41 | 0 | 0 | 0 | 0 | 0 | 113 | 37 | 0 |
| Dichapetalaceae | 0 | 0 | 0 | 0 | 0 | 0 | 0 | 24 | 0 | 0 |
| Dilleniaceae | 0 | 0 | 0 | 0 | 17 | 0 | 202 | 522 | 0 | 0 |
| Dioscoreaceae | 6 | 15 | 0 | 67 | 82 | 0 | 15 | 125 | 15 | 15 |
| Droseraceae | 0 | 0 | 0 | 0 | 0 | 0 | 0 | 31 | 0 | 0 |
| Ebenaceae | 0 | 0 | 0 | 0 | 8 | 0 | 15 | 13 | 0 | 0 |
| Elaeocarpaceae | 0 | 0 | 0 | 0 | 0 | 0 | 505 | 33 | 0 | 0 |
| Equisetaceae | 0 | 0 | 0 | 0 | 0 | 0 | 0 | 408 | 0 | 0 |
| Ericaceae | 0 | 1644 | 0 | 7 | 2042 | 0 | 517 | 3636 | 0 | 0 |
| Eriocaulaceae | 0 | 0 | 0 | 0 | 0 | 0 | 0 | 13 | 0 | 0 |
| Erythroxylaceae | 0 | 110 | 0 | 0 | 80 | 0 | 288 | 400 | 30 | 110 |
| Escalloniaceae | 0 | 0 | 0 | 0 | 0 | 0 | 19 | 0 | 0 | 0 |
| Euphorbiaceae | 1224 | 2494 | 1133 | 1139 | 1971 | 752 | 3605 | 5034 | 2034 | 1221 |
| Fabaceae | 6954 | 9595 | 2482 | 3572 | 7334 | 2004 | 9134 | 14732 | 4712 | 1649 |
| Fagaceae | 0 | 0 | 0 | 0 | 0 | 0 | 877 | 0 | 0 | 0 |
| Gentianaceae | 0 | 2 | 0 | 0 | 21 | 0 | 0 | 403 | 0 | 0 |
| Geraniaceae | 0 | 34 | 0 | 22 | 7 | 0 | 19 | 105 | 14 | 0 |
| Gesneriaceae | 0 | 1072 | 0 | 0 | 0 | 0 | 0 | 1353 | 0 | 0 |
| Gleicheniaceae | 0 | 0 | 0 | 0 | 0 | 0 | 0 | 84 | 0 | 0 |
| Goupiaceae | 0 | 0 | 0 | 0 | 0 | 0 | 184 | 184 | 0 | 0 |
| Gunneraceae | 0 | 0 | 0 | 0 | 0 | 0 | 0 | 49 | 0 | 0 |
| Haemodoraceae | 0 | 0 | 0 | 0 | 0 | 0 | 0 | 359 | 0 | 0 |
| Haloragaceae | 0 | 15 | 0 | 0 | 15 | 0 | 0 | 0 | 0 | 0 |
| Heliconiaceae | 0 | 842 | 0 | 70 | 0 | 0 | 99 | 211 | 0 | 29 |
| Hernandiaceae | 0 | 0 | 49 | 0 | 0 | 0 | 70 | 63 | 0 | 0 |
| Humiriaceae | 0 | 0 | 0 | 0 | 124 | 0 | 36 | 26 | 0 | 0 |
| Hydrangeaceae | 0 | 29 | 0 | 0 | 0 | 0 | 0 | 29 | 29 | 0 |
| Hydrocharitaceae | 1 | 16 | 0 | 0 | 0 | 0 | 0 | 1 | 0 | 0 |
| Hydroleaceae | 0 | 0 | 0 | 0 | 0 | 0 | 0 | 42 | 0 | 0 |
| Hymenophyllaceae | 0 | 0 | 0 | 0 | 0 | 0 | 0 | 348 | 0 | 0 |
| Hypericaceae | 0 | 3 | 0 | 0 | 0 | 0 | 553 | 1083 | 0 | 0 |
| Hypoxidaceae | 0 | 0 | 0 | 0 | 0 | 0 | 0 | 10 | 0 | 0 |
| Iridaceae | 0 | 256 | 0 | 0 | 0 | 0 | 0 | 106 | 22 | 0 |
| Juglandaceae | 0 | 101 | 0 | 101 | 101 | 0 | 101 | 101 | 0 | 0 |
| Juncaceae | 195 | 195 | 139 | 139 | 196 | 0 | 139 | 236 | 139 | 0 |
| Krameriaceae | 0 | 0 | 0 | 0 | 0 | 0 | 0 | 3 | 0 | 0 |
| Lacistemataceae | 0 | 0 | 0 | 0 | 0 | 0 | 0 | 393 | 0 | 0 |
| Lamiaceae | 166 | 499 | 49 | 96 | 711 | 91 | 517 | 2212 | 129 | 224 |
| Lauraceae | 210 | 213 | 213 | 382 | 376 | 210 | 1714 | 1420 | 265 | 265 |
| Lecythidaceae | 0 | 86 | 23 | 3 | 216 | 0 | 826 | 427 | 0 | 0 |
| Liliaceae | 0 | 3 | 0 | 0 | 0 | 0 | 0 | 3 | 0 | 0 |
| Linaceae | 21 | 0 | 21 | 0 | 21 | 0 | 21 | 72 | 21 | 0 |
| Linderniaceae | 0 | 9 | 0 | 0 | 0 | 0 | 0 | 234 | 0 | 0 |
| Loasaceae | 0 | 0 | 0 | 0 | 0 | 0 | 0 | 7 | 0 | 0 |
| Loganiaceae | 49 | 0 | 0 | 0 | 49 | 0 | 120 | 344 | 96 | 49 |
| Loranthaceae | 0 | 0 | 0 | 0 | 0 | 0 | 95 | 1489 | 0 | 0 |
| Lycopodiaceae | 809 | 809 | 809 | 0 | 809 | 0 | 809 | 814 | 0 | 809 |
| Lythraceae | 28 | 117 | 146 | 21 | 45 | 12 | 267 | 687 | 45 | 45 |
| Magnoliaceae | 0 | 0 | 0 | 0 | 0 | 0 | 16 | 0 | 0 | 0 |
| Malpighiaceae | 298 | 538 | 298 | 16 | 688 | 298 | 546 | 1084 | 0 | 36 |
| Malvaceae | 1534 | 2031 | 1244 | 1932 | 2111 | 941 | 3363 | 4520 | 887 | 1804 |
| Marantaceae | 42 | 42 | 0 | 32 | 49 | 0 | 261 | 175 | 32 | 32 |
| Marcgraviaceae | 0 | 0 | 0 | 0 | 0 | 0 | 64 | 148 | 0 | 0 |
| Marsileaceae | 0 | 0 | 0 | 0 | 0 | 0 | 0 | 21 | 0 | 0 |
| Martyniaceae | 0 | 0 | 0 | 0 | 2 | 0 | 0 | 2 | 0 | 0 |
| Melastomataceae | 0 | 301 | 0 | 300 | 1070 | 0 | 1861 | 2165 | 0 | 0 |
| Meliaceae | 97 | 384 | 294 | 83 | 384 | 283 | 2115 | 1966 | 522 | 83 |
| Menispermaceae | 93 | 93 | 0 | 0 | 93 | 0 | 241 | 509 | 93 | 0 |
| Menyanthaceae | 0 | 43 | 0 | 43 | 43 | 43 | 43 | 43 | 0 | 0 |
| Metteniusaceae | 0 | 0 | 0 | 0 | 82 | 0 | 167 | 78 | 0 | 0 |
| Microteaceae | 0 | 0 | 0 | 0 | 0 | 0 | 0 | 30 | 0 | 0 |
| Molluginaceae | 0 | 0 | 0 | 0 | 0 | 0 | 0 | 25 | 0 | 0 |
| Montiaceae | 0 | 0 | 0 | 0 | 3 | 0 | 0 | 0 | 0 | 0 |
| Moraceae | 399 | 383 | 129 | 138 | 1299 | 68 | 2522 | 4011 | 368 | 429 |
| Moringaceae | 7 | 7 | 7 | 7 | 7 | 7 | 7 | 7 | 7 | 7 |
| Muntingiaceae | 0 | 191 | 191 | 0 | 191 | 0 | 197 | 191 | 0 | 0 |
| Musaceae | 28 | 47 | 0 | 71 | 28 | 4 | 39 | 71 | 0 | 24 |
| Myricaceae | 0 | 0 | 0 | 0 | 0 | 0 | 229 | 315 | 0 | 0 |
| Myristicaceae | 0 | 0 | 0 | 0 | 272 | 0 | 1011 | 1665 | 0 | 0 |
| Myrtaceae | 318 | 707 | 501 | 400 | 1153 | 303 | 1618 | 2207 | 402 | 39 |
| Nelumbonaceae | 0 | 0 | 0 | 0 | 0 | 0 | 0 | 3 | 0 | 0 |
| Nyctaginaceae | 56 | 224 | 0 | 18 | 115 | 0 | 229 | 332 | 71 | 71 |
| Nymphaeaceae | 5 | 18 | 0 | 5 | 15 | 0 | 8 | 32 | 0 | 5 |
| Ochnaceae | 0 | 0 | 0 | 0 | 0 | 0 | 361 | 381 | 36 | 0 |
| Olacaceae | 6 | 6 | 6 | 6 | 6 | 0 | 113 | 358 | 6 | 6 |
| Oleaceae | 12 | 35 | 6 | 6 | 23 | 6 | 41 | 35 | 5 | 9 |
| Onagraceae | 303 | 388 | 0 | 50 | 87 | 0 | 318 | 774 | 37 | 158 |
| Ophioglossaceae | 0 | 0 | 0 | 0 | 0 | 0 | 0 | 25 | 0 | 0 |
| Opiliaceae | 0 | 0 | 0 | 0 | 0 | 0 | 15 | 0 | 0 | 0 |
| Orchidaceae | 0 | 522 | 0 | 79 | 52 | 0 | 67 | 933 | 0 | 0 |
| Orobanchaceae | 0 | 0 | 0 | 0 | 76 | 0 | 76 | 218 | 10 | 0 |
| Osmundaceae | 0 | 25 | 0 | 16 | 16 | 0 | 16 | 25 | 16 | 0 |
| Oxalidaceae | 113 | 141 | 0 | 113 | 164 | 79 | 113 | 270 | 124 | 79 |
| Pandanaceae | 1 | 1 | 0 | 0 | 1 | 0 | 1 | 1 | 0 | 1 |
| Papaveraceae | 19 | 144 | 19 | 9 | 28 | 0 | 137 | 184 | 35 | 28 |
| Passifloraceae | 348 | 1746 | 0 | 1077 | 1267 | 240 | 454 | 2388 | 461 | 194 |
| Pentaphylacaceae | 0 | 0 | 0 | 0 | 0 | 0 | 38 | 0 | 0 | 0 |
| Peraceae | 0 | 0 | 0 | 0 | 0 | 0 | 15 | 0 | 0 | 0 |
| Petiveriaceae | 70 | 70 | 0 | 0 | 122 | 70 | 53 | 254 | 192 | 122 |
| Phrymaceae | 0 | 0 | 0 | 0 | 0 | 0 | 0 | 15 | 0 | 0 |
| Phyllanthaceae | 113 | 74 | 11 | 11 | 93 | 0 | 538 | 734 | 161 | 0 |
| Phytolaccaceae | 10 | 26 | 0 | 26 | 347 | 0 | 10 | 619 | 64 | 0 |
| Picramniaceae | 0 | 0 | 0 | 0 | 0 | 0 | 22 | 22 | 0 | 0 |
| Pinaceae | 0 | 55 | 42 | 6 | 55 | 0 | 55 | 55 | 0 | 0 |
| Piperaceae | 454 | 659 | 453 | 177 | 1012 | 0 | 1131 | 3211 | 1 | 0 |
| Pittosporaceae | 0 | 22 | 0 | 0 | 0 | 0 | 22 | 0 | 0 | 0 |
| Plantaginaceae | 339 | 300 | 0 | 104 | 339 | 0 | 480 | 913 | 386 | 245 |
| Plumbaginaceae | 10 | 23 | 0 | 4 | 13 | 0 | 19 | 19 | 19 | 10 |
| Poaceae | 2885 | 2276 | 502 | 1359 | 1184 | 425 | 2839 | 3738 | 1157 | 990 |
| Podocarpaceae | 0 | 0 | 0 | 0 | 0 | 0 | 217 | 0 | 0 | 0 |
| Polemoniaceae | 0 | 23 | 0 | 0 | 0 | 0 | 0 | 20 | 0 | 0 |
| Polygalaceae | 0 | 0 | 0 | 0 | 109 | 0 | 28 | 281 | 0 | 109 |
| Polygonaceae | 51 | 70 | 15 | 118 | 198 | 0 | 403 | 771 | 176 | 1 |
| Polypodiaceae | 0 | 731 | 0 | 36 | 36 | 0 | 108 | 1336 | 186 | 149 |
| Pontederiaceae | 0 | 27 | 0 | 0 | 85 | 0 | 85 | 112 | 15 | 0 |
| Portulacaceae | 81 | 111 | 0 | 81 | 111 | 0 | 81 | 112 | 81 | 81 |
| Potamogetonaceae | 0 | 5 | 0 | 0 | 0 | 0 | 0 | 3 | 0 | 0 |
| Primulaceae | 0 | 49 | 0 | 0 | 17 | 0 | 1239 | 1390 | 1 | 0 |
| Proteaceae | 8 | 8 | 5 | 8 | 8 | 5 | 139 | 94 | 5 | 0 |
| Psilotaceae | 0 | 1 | 0 | 0 | 0 | 1 | 0 | 1 | 0 | 0 |
| Pteridaceae | 30 | 509 | 0 | 34 | 34 | 0 | 246 | 846 | 219 | 216 |
| Ranunculaceae | 0 | 2 | 0 | 0 | 0 | 0 | 0 | 86 | 0 | 0 |
| Rhamnaceae | 16 | 7 | 16 | 3 | 97 | 79 | 97 | 122 | 3 | 3 |
| Rhizophoraceae | 0 | 0 | 34 | 34 | 34 | 0 | 74 | 34 | 0 | 0 |
| Rosaceae | 94 | 178 | 65 | 159 | 354 | 17 | 160 | 901 | 95 | 74 |
| Rubiaceae | 1024 | 1475 | 23 | 466 | 1616 | 685 | 3318 | 10160 | 582 | 1038 |
| Ruppiaceae | 0 | 2 | 0 | 0 | 0 | 0 | 0 | 2 | 0 | 0 |
| Rutaceae | 10 | 126 | 10 | 192 | 123 | 42 | 605 | 658 | 71 | 113 |
| Salicaceae | 1 | 77 | 1 | 2 | 6 | 0 | 1132 | 1411 | 512 | 0 |
| Salviniaceae | 50 | 93 | 0 | 0 | 0 | 0 | 0 | 50 | 0 | 0 |
| Santalaceae | 0 | 0 | 0 | 0 | 0 | 0 | 0 | 234 | 0 | 0 |
| Sapindaceae | 305 | 1018 | 277 | 666 | 1137 | 183 | 1428 | 1596 | 510 | 379 |
| Sapotaceae | 0 | 59 | 22 | 59 | 320 | 0 | 976 | 795 | 0 | 59 |
| Schizaeaceae | 0 | 47 | 0 | 0 | 0 | 0 | 12 | 147 | 0 | 0 |
| Schlegeliaceae | 0 | 0 | 0 | 0 | 0 | 0 | 42 | 1 | 0 | 0 |
| Scrophulariaceae | 1 | 251 | 69 | 0 | 38 | 0 | 107 | 289 | 0 | 0 |
| Selaginellaceae | 2 | 44 | 0 | 0 | 3 | 0 | 2 | 426 | 0 | 0 |
| Simaroubaceae | 221 | 252 | 221 | 0 | 260 | 0 | 380 | 312 | 260 | 0 |
| Siparunaceae | 0 | 0 | 0 | 0 | 0 | 0 | 161 | 844 | 0 | 0 |
| Smilacaceae | 0 | 0 | 0 | 0 | 0 | 0 | 9 | 153 | 0 | 0 |
| Solanaceae | 613 | 2324 | 30 | 2943 | 1946 | 182 | 1943 | 5011 | 1630 | 1014 |
| Sphenocleaceae | 53 | 0 | 0 | 0 | 53 | 0 | 0 | 53 | 0 | 0 |
| Staphyleaceae | 0 | 0 | 0 | 0 | 0 | 0 | 214 | 214 | 0 | 0 |
| Strelitziaceae | 3 | 7 | 0 | 3 | 7 | 0 | 50 | 7 | 0 | 0 |
| Styracaceae | 0 | 0 | 0 | 0 | 0 | 0 | 1 | 0 | 0 | 0 |
| Surianaceae | 0 | 3 | 0 | 0 | 0 | 0 | 3 | 3 | 0 | 0 |
| Symplocaceae | 0 | 0 | 0 | 0 | 151 | 0 | 0 | 151 | 0 | 0 |
| Talinaceae | 46 | 94 | 0 | 94 | 94 | 0 | 46 | 94 | 46 | 46 |
| Theaceae | 0 | 10 | 0 | 16 | 10 | 0 | 147 | 10 | 0 | 0 |
| Thymelaeaceae | 0 | 0 | 0 | 0 | 0 | 0 | 0 | 46 | 0 | 0 |
| Tropaeolaceae | 0 | 60 | 0 | 45 | 59 | 0 | 45 | 59 | 0 | 0 |
| Typhaceae | 17 | 17 | 4 | 17 | 17 | 0 | 17 | 17 | 13 | 4 |
| Ulmaceae | 0 | 0 | 0 | 0 | 0 | 0 | 87 | 0 | 0 | 0 |
| Urticaceae | 169 | 84 | 413 | 30 | 893 | 0 | 919 | 1495 | 403 | 30 |
| Verbenaceae | 513 | 871 | 514 | 535 | 778 | 366 | 780 | 1328 | 403 | 696 |
| Viburnaceae | 0 | 53 | 0 | 0 | 53 | 0 | 53 | 246 | 53 | 0 |
| Violaceae | 0 | 34 | 0 | 0 | 34 | 0 | 105 | 124 | 0 | 0 |
| Vitaceae | 10 | 211 | 10 | 71 | 64 | 0 | 232 | 563 | 0 | 10 |
| Vochysiaceae | 0 | 0 | 0 | 0 | 12 | 0 | 378 | 125 | 0 | 0 |
| Winteraceae | 0 | 0 | 0 | 0 | 0 | 0 | 0 | 428 | 0 | 0 |
| Xyridaceae | 0 | 0 | 0 | 0 | 0 | 0 | 0 | 115 | 0 | 0 |
| Zamiaceae | 0 | 15 | 0 | 0 | 0 | 0 | 0 | 0 | 0 | 0 |
| Zingiberaceae | 44 | 222 | 3 | 61 | 312 | 3 | 272 | 536 | 15 | 44 |
| Zygophyllaceae | 0 | 15 | 0 | 16 | 31 | 15 | 86 | 63 | 1 | 0 |

**S2.9 Table. All vascular plants: surveyed grid cells in bioregions.**

| **Bioregion** | | **Total number of 10x10 km grid cells (study area)** | **Surveyed grid cells** | | **Well surveyed grid cells (>= 25 observations)** | |
| --- | --- | --- | --- | --- | --- | --- |
| **ID** | **Name** |  | **Number** | **% of the Total** | **Number** | **% of the Surveyed** |
| **3** | **Paramos - Andes** | 133 | 120 | 90.23 | 83 | 69.17 |
| **10** | **Moist forests - Andes** | 1521 | 1100 | 72.32 | 570 | 51.82 |
| **5** | **Dry forests - Andes** | 95 | 85 | 89.47 | 44 | 51.76 |
| **2** | **Mangroves** | 77 | 32 | 41.56 | 15 | 46.88 |
| **14** | **Transformed areas** | 3584 | 2640 | 73.66 | 1202 | 45.53 |
| **13** | **Moist forests - Pacifico** | 559 | 318 | 56.89 | 139 | 43.71 |
| **12** | **Moist forests - Llanos** | 1374 | 363 | 26.42 | 127 | 34.99 |
| **9** | **Moist forests - Amazonas** | 3162 | 953 | 30.14 | 329 | 34.52 |
| **11** | **Moist forests - Caribe** | 441 | 227 | 51.47 | 78 | 34.36 |
| **6** | **Dry forests - Llanos** | 134 | 69 | 51.49 | 21 | 30.43 |
| **1** | **Deserts and xeric shrublands - Caribe** | 201 | 91 | 45.27 | 26 | 28.57 |
| **7** | **Dry forests - Caribe** | 77 | 42 | 54.55 | 12 | 28.57 |
| **4** | **Paramos - Caribe** | 17 | 17 | 100.00 | 4 | 23.53 |
| **8** | **Savannas - Llanos** | 1535 | 494 | 32.18 | 112 | 22.67 |

**S2.10 Table. Useful plants: surveyed grid cells in bioregions.**

| **Bioregion** | | **Total number of 10x10 km grid cells (study area)** | **Surveyed grid cells** | | **Well surveyed grid cells (>= 25 observations)** | |
| --- | --- | --- | --- | --- | --- | --- |
| **ID** | **Name** |  | **Number** | **% of the Total** | **Number** | **% of the Surveyed** |
| 3 | **Paramos - Andes** | 133 | 105 | 78.95 | 55 | 52.38 |
| 5 | **Dry forests - Andes** | 95 | 83 | 87.37 | 34 | 40.96 |
| 10 | **Moist forests - Andes** | 1521 | 970 | 63.77 | 343 | 35.36 |
| 14 | **Transformed areas** | 3584 | 2433 | 67.89 | 803 | 33.00 |
| 13 | **Moist forests - Pacifico** | 559 | 274 | 49.02 | 86 | 31.39 |
| 12 | **Moist forests - Llanos** | 1374 | 295 | 21.47 | 84 | 28.47 |
| 6 | **Dry forests - Llanos** | 134 | 64 | 47.76 | 15 | 23.44 |
| 9 | **Moist forests - Amazonas** | 3162 | 764 | 24.16 | 176 | 23.04 |
| 11 | **Moist forests - Caribe** | 441 | 196 | 44.44 | 44 | 22.45 |
| 2 | **Mangroves** | 77 | 32 | 41.56 | 7 | 21.88 |
| 1 | **Deserts and xeric shrublands - Caribe** | 201 | 79 | 39.30 | 16 | 20.25 |
| 7 | **Dry forests - Caribe** | 77 | 39 | 50.65 | 7 | 17.95 |
| 8 | **Savannas - Llanos** | 1535 | 404 | 26.32 | 66 | 16.34 |
| 4 | **Paramos - Caribe** | 17 | 12 | 70.59 | 1 | 8.33 |

**
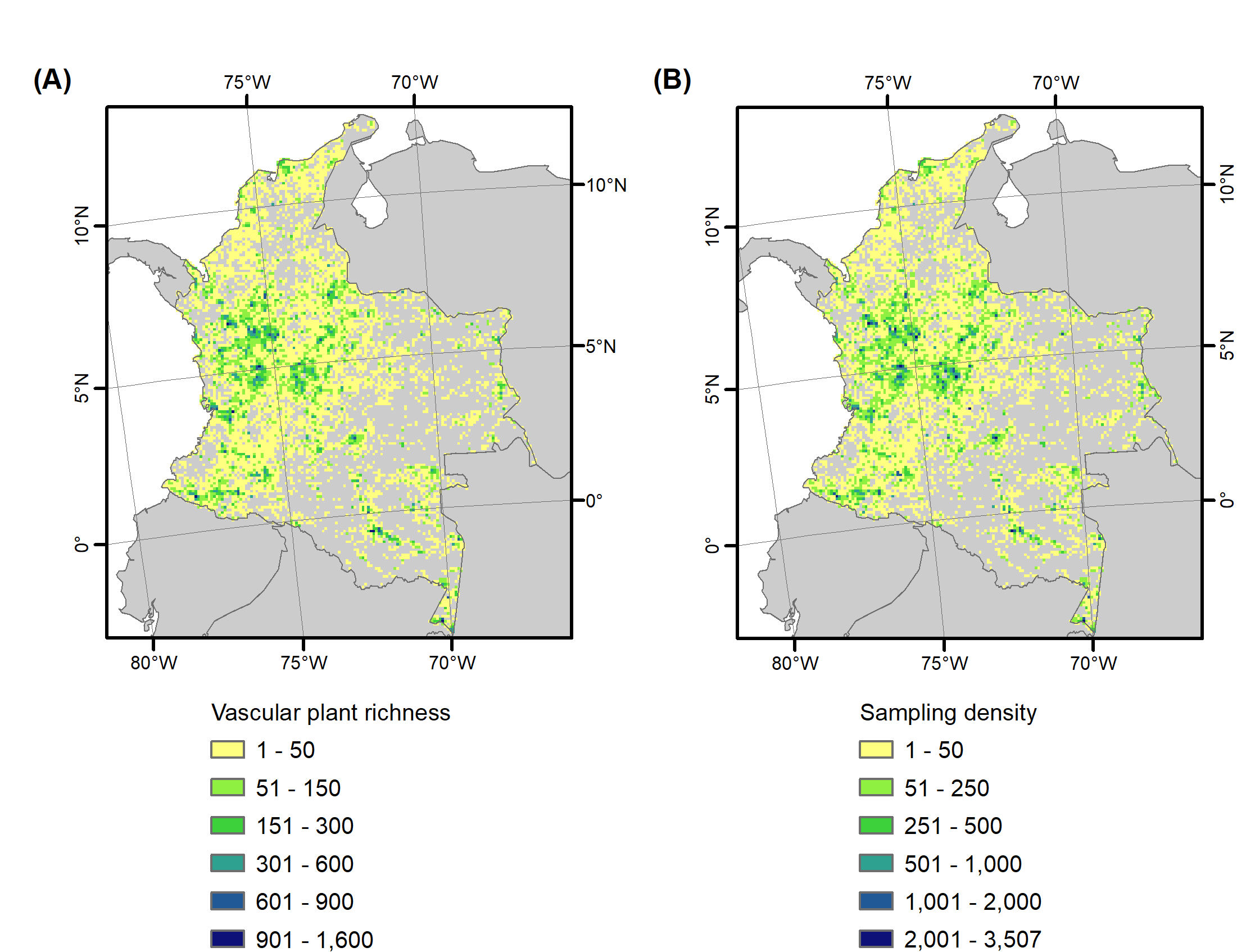
**

S2.1 Fig. All vascular plant species richness (A) and samples (B).


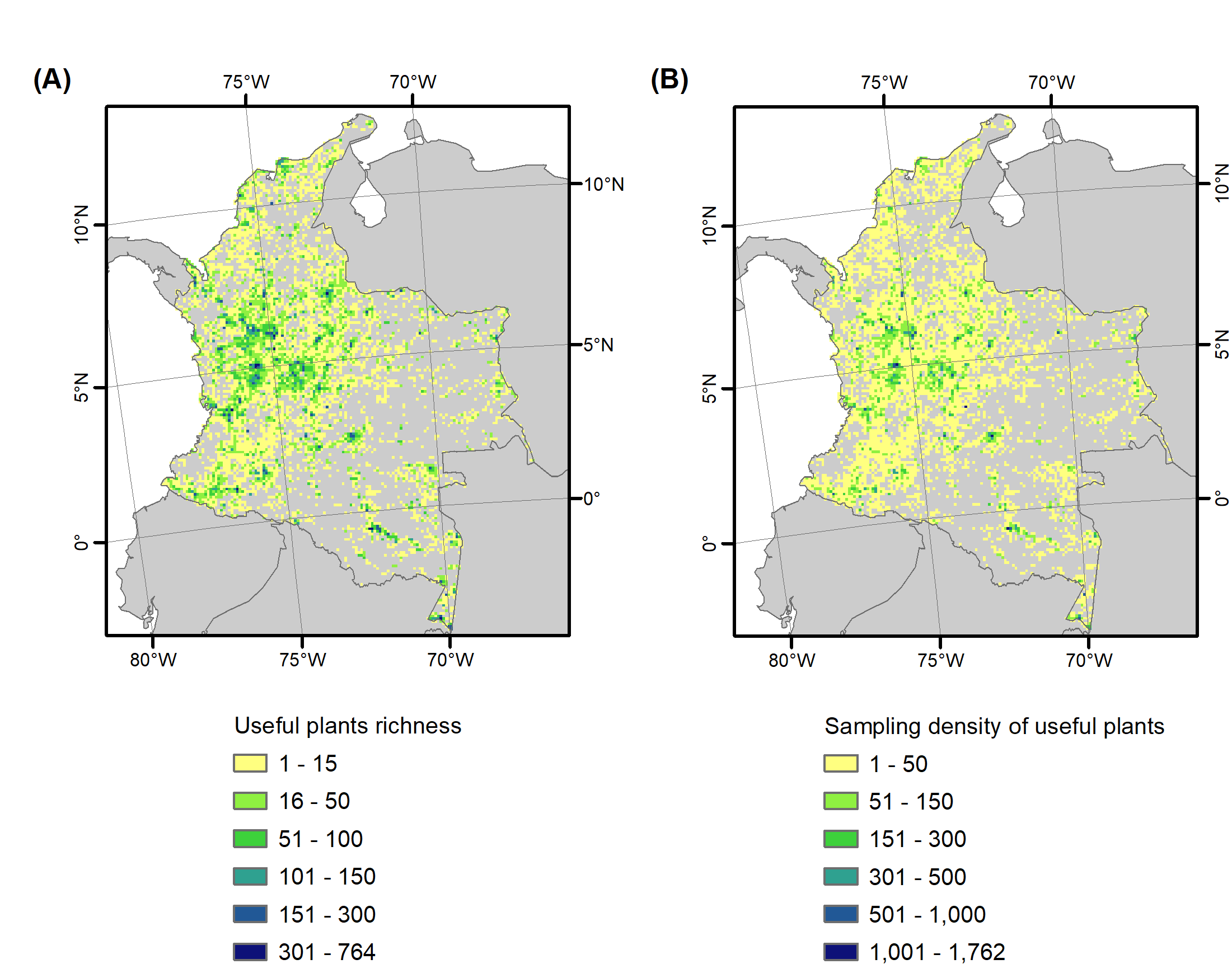


S2.2 Fig. Useful plant species richness (A) and samples (B).


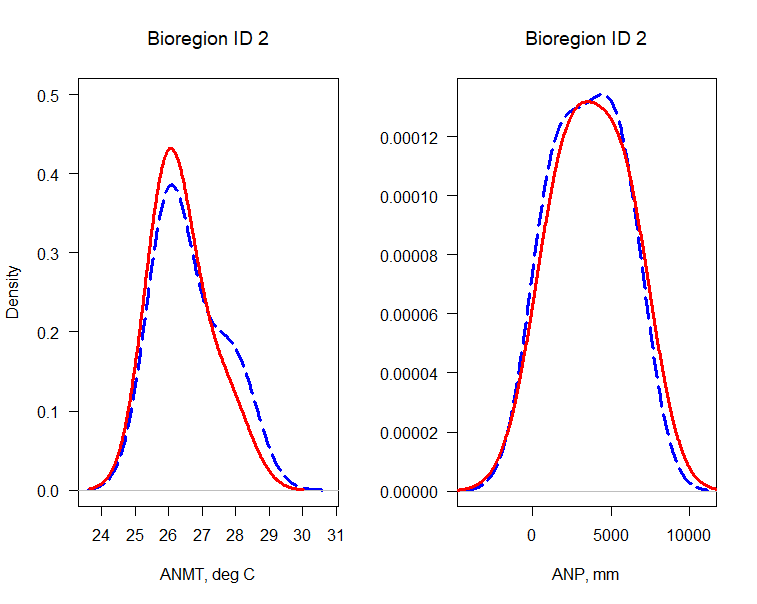

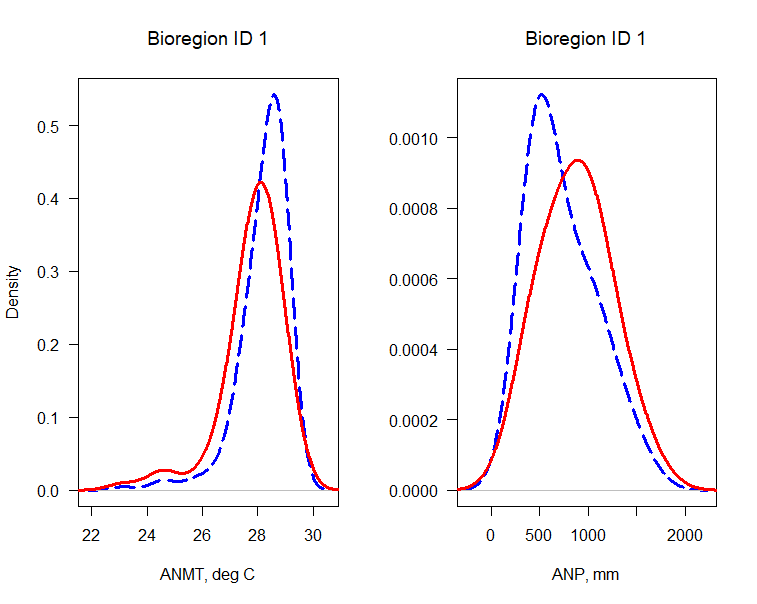

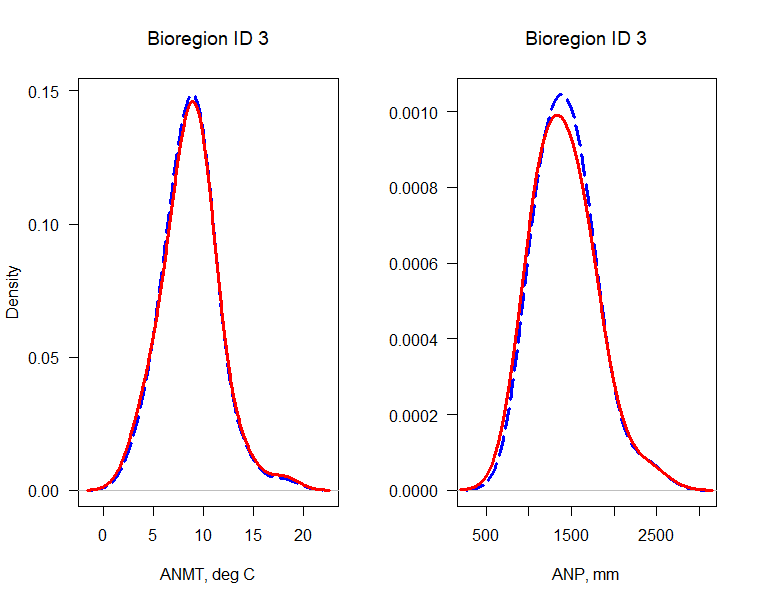

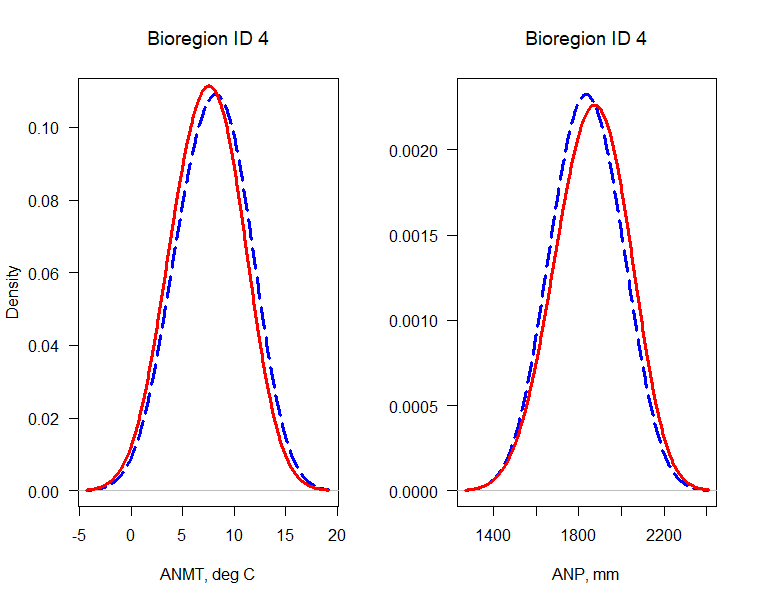

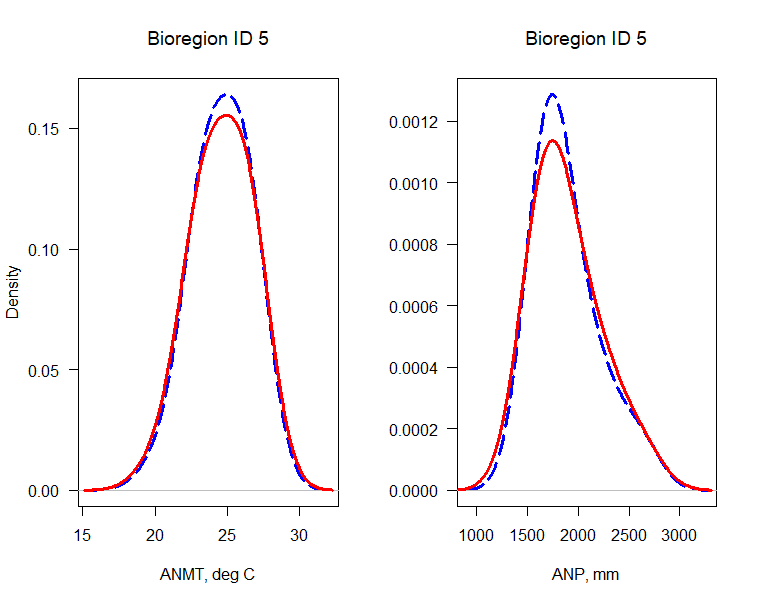

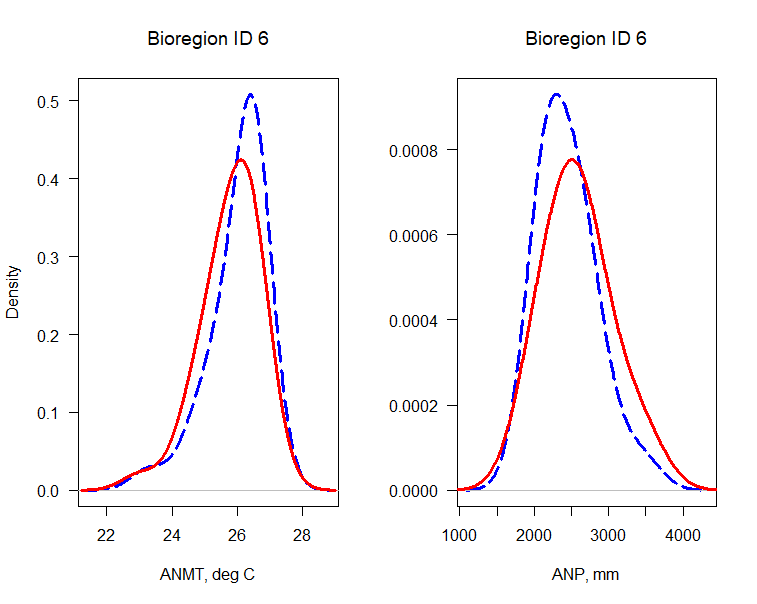


S2.3 Fig. Probability density functions (PDFs) have been estimated along annual mean temperature (ANMT, left column) and annual precipitation (ANP, right column) in bioregions 1 – 6. Solid line: distribution of grid cells with records of useful plants; dashed line: distribution of cells in the whole bioregion. ID 1: Caribbean deserts and xeric shrublands; ID 2: Mangroves; ID 3: Andean paramo; ID 4: Caribbean paramo; ID 5: Andean dry forest; ID 6: Llanos dry forest.


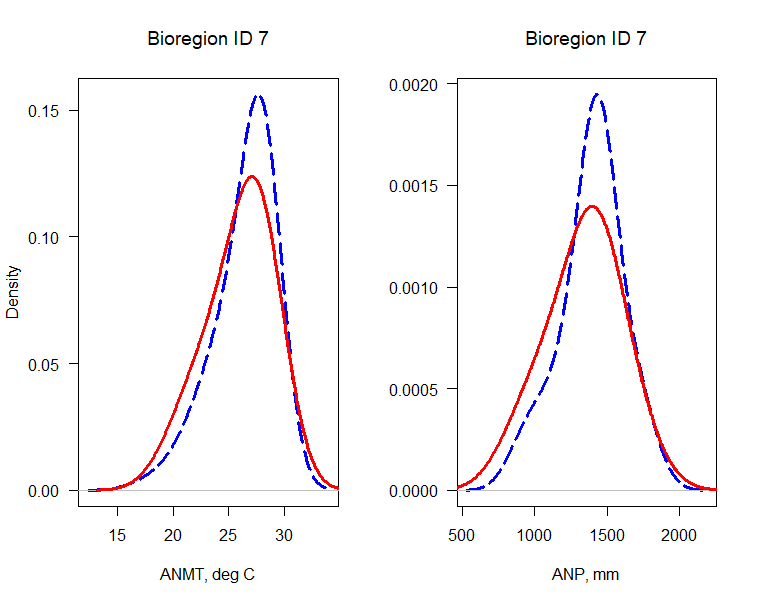

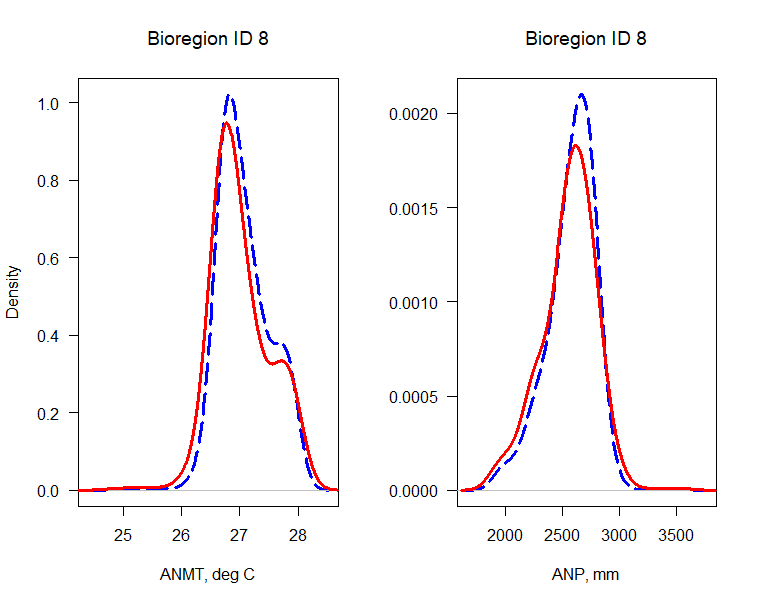

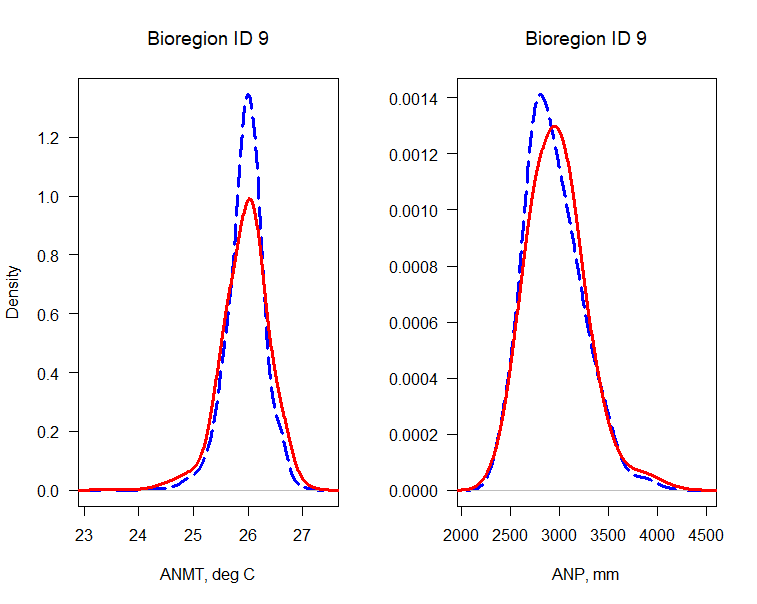

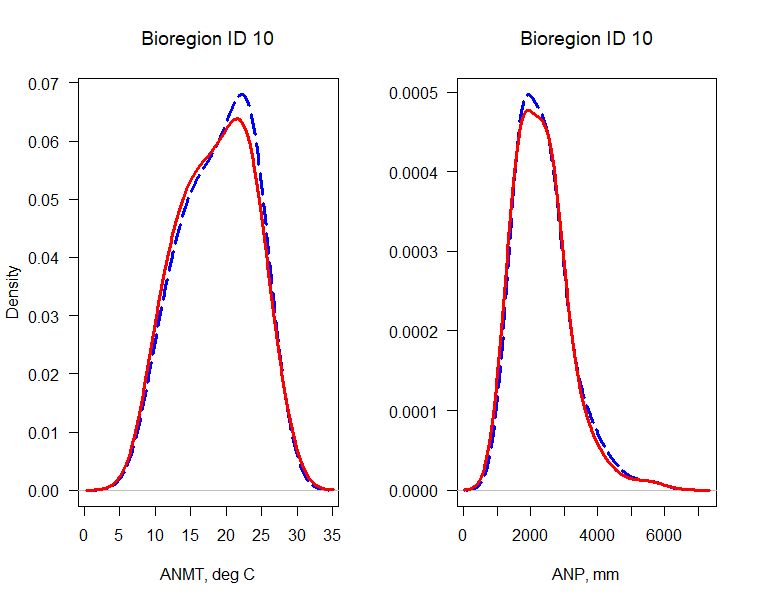

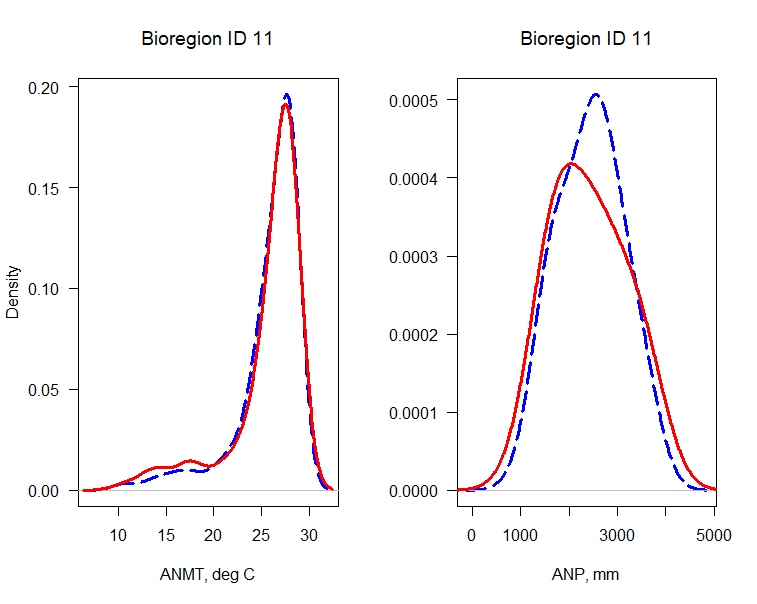

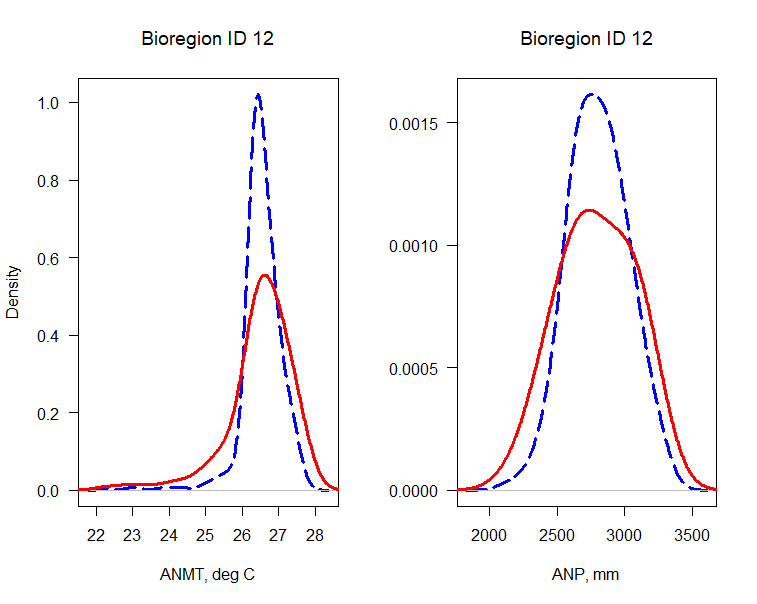


S2.4 Fig. Probability density functions (PDFs) have been estimated along annual mean temperature (ANMT, left column) and annual precipitation (ANP, right column) in bioregions 7 – 12. Solid line: distribution of grid cells with records of useful plants; dashed line: distribution of cells in the whole bioregion. ID 7: Caribbean dry forest; ID 8: Savanna; ID 9: Amazonian moist forest; ID 10: Andean moist forest; ID 11: Caribbean moist forest; ID 12: Llanos moist forest.


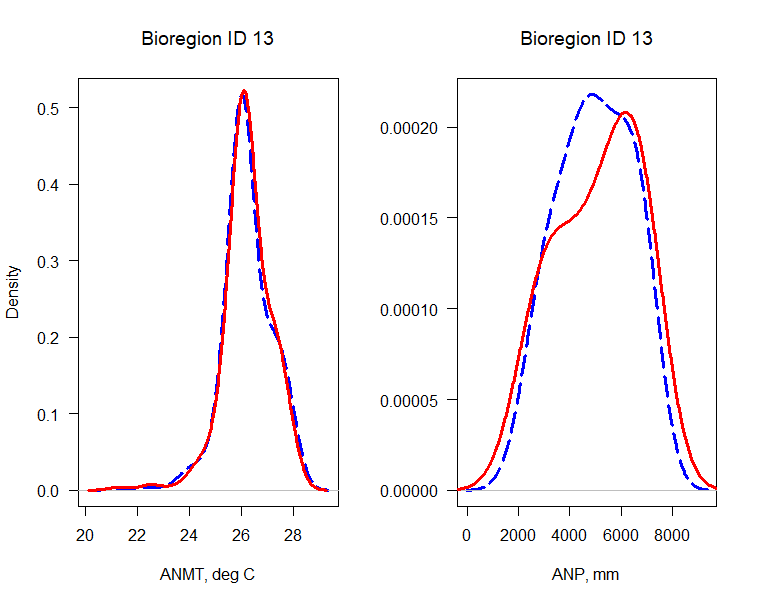

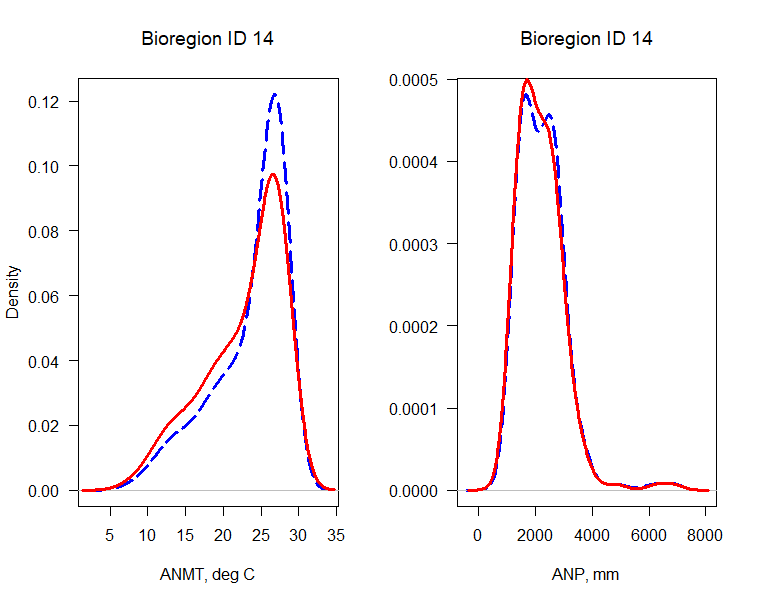


S2.5 Fig. Probability density functions (PDFs) have been estimated along annual mean temperature (ANMT, left column) and annual precipitation (ANP, right column) in bioregions 13 and 14: Pacific moist forest and transformed areas. Solid line: distribution of grid cells with records of useful plants; dashed line: distribution of cells in the whole bioregion.


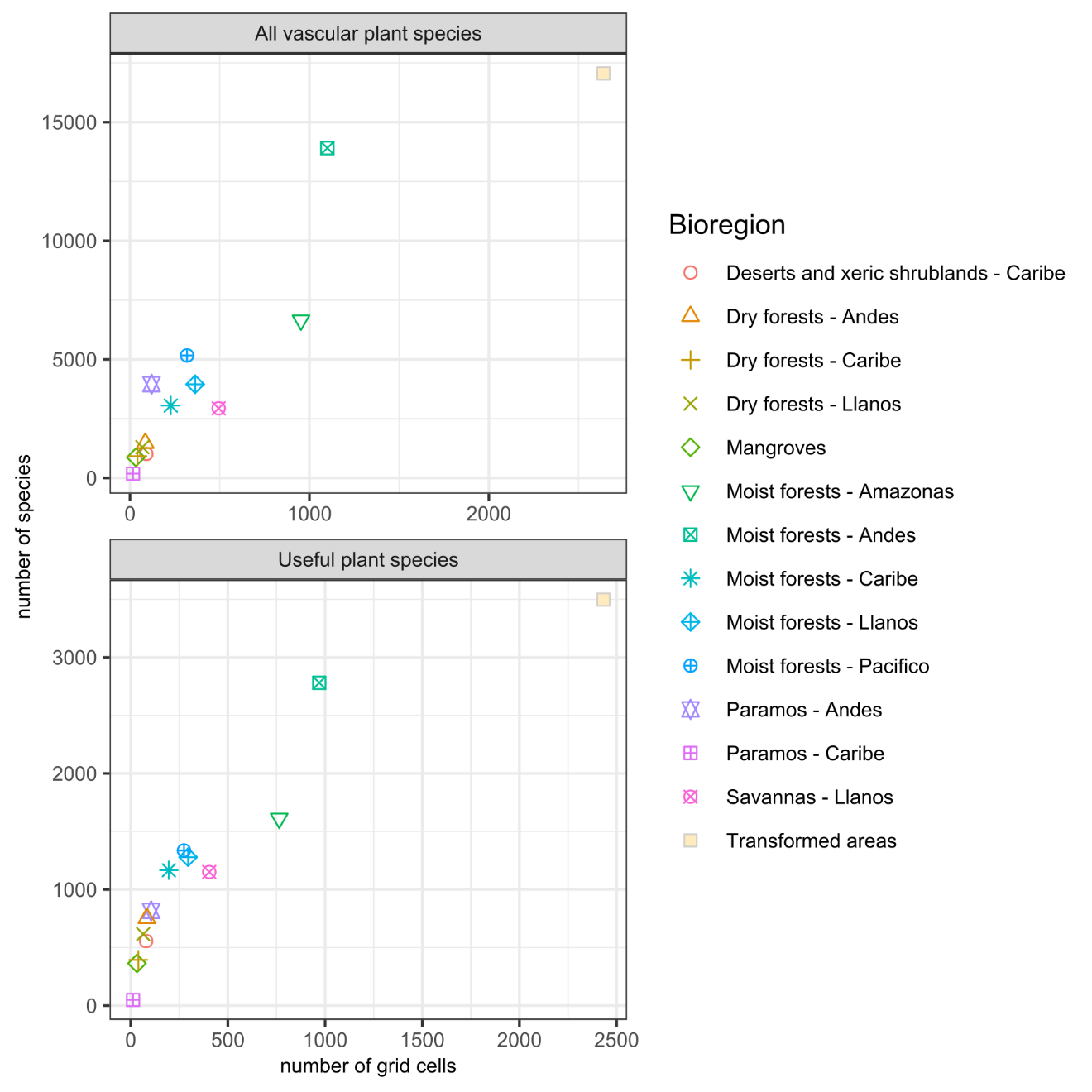


S2.6 Fig. Scatterplots comparing all vascular plant and useful plant species numbers to numbers of sampled grid cells within each bioregion.
