# Supplementary material for: Colombia’s bioregions as a source of useful plants
Source: PLoS One. 2021 Aug 27;16(8):e0256457. doi: 10.1371/journal.pone.0256457 (PMC8396733; doi:10.1371/journal.pone.0256457)
Supplement: Attachment — Submitted filename: Comments@PLOS ONE.docx [file pone.0256457.s003.docx]

**Comments:**

**General comments:**

In the work “Colombia's bioregions as a source of useful plants”, the authors assembled a dataset of georeferenced collection localities of all vascular plants and useful plants of Colombia, and put all point locality information in a map of Colombia’s bioregions, and they found Andean, Amazon, Pacific, Llanos and Caribbean moist forests have the highest numbers of all vascular plant species and useful species descendingly. The manuscript is well written and data analysis is supportive of their conclusions. I think, with more work like this, may be conducted in other areas in the world, we will have a more specific layout of the useful plants and detailed conservation plans of the plant species in the Anthropocene during which nearly all the plant species face a high extinction risk.

I am very happy to see such a work be published if they could address my following concerns:

1) It’s important to stress the importance of this or this kind of work. Collecting the information of useful plants in a specific area and showing the results publicly, I think, one of (those) aims to protect or conserve these valuable plant species, which should be addressed in the Introduction and Discussion. The authors may find it useful as a reference (Gao et al, Plant extinction excels plant speciation in the Anthropocene. *BMC Plant Biology* 2020, 20: 430. https://doi.org/10.1186/s12870-020-02646-3).

2) The authors said “traditional knowledge and the importance of medicinal plants in Colombia that dates back to the last 30 years” and “Colombia ethnobotanical studies on indigenous populations began in 1860 with the work of Florentino Vezga”, which probably not the truth. As I knew, Alexander von Humboldt has traveled to South America (or Colombia) and collected many useful plants and carried them to Europe which was definitely before 1860 (which highlighted in their Acknowledgements). The authors may find it useful to read Wulf’s book (The invention of nature: The adventures of Alexander von Humboldt, the lost hero of science (John Murray, London, UK, 2015)).

3) The authors explained the biodiversity of Pacific moist forests that “might have developed only in the last 5 million years once the landscape had changed into fully terrestrial”, which is very interesting. Can you expand it? (Including other bioregions of their manuscript) Linking plant evolutionary history and plant use is very crucial for our understanding of the current use scenarios of many plants. The authors may find it useful to refer Molina-Venegas’s recent work (Maximum levels of global phylogenetic diversity efficiently capture plant services for humankind. https://doi.org/10.1038/s41559-021-01414-2).

4) The authors indeed discussed their survey completeness and survey coverage of the useful plants, but not highlighted in the Discussion fully. The authors may find it useful to refer to a similar work “Ethnobotanical Knowledge Is Vastly Under-Documented in Northwestern South America” by Camara-Leret et al. (doi: 10.1371/journal.pone.0085794).

5) I strongly suggested moving Table S2.2 (Categories of plant uses following Diazgranados et al. (2020)) and Table S2.3 (Useful plants and their uses) to the main text. Because they are very crucial to understand some definitions used in this study. And one thing you should keep in mind, some plants are used both as Medicines and Human Food, or tri-usefulness (three or even more uses), which may be explained in the table caption.

6) In the discussion, they highlighted the importance of useful plants in promoting a bio-economy (reference 36 they cited), but the readers may be interested in the applications of these useful plants in achieving UN Sustainable Development Goals, especially in the face of COVID-19. For me, the useful plants in Colombia are key, at least, to some extent, in achieving Sustainable Development Goals 1 (No Poverty), 12 (Responsible Consumption and Production), 13 (Climate Action), and 15 (Life on Land). Can you expand it?

**Minor comments:**

Line 29: Formatting issue: gaps-,.

Line 203: Use abbreviations because you have already defined them (Line 157: annual mean temperature (ANMT) and total annual precipitation (ANP)).

Line 320: According to what? I believe you gave such an exact number (70%) must have based on some empirical study.

Line 436: 3870/4200 is a high proportion, not “only”.
