# Supplementary material for: Colombia’s bioregions as a source of useful plants
Source: PLoS One. 2021 Aug 27;16(8):e0256457. doi: 10.1371/journal.pone.0256457 (PMC8396733; doi:10.1371/journal.pone.0256457)
Supplement: Attachment — Submitted filename: Response to Reviewers.docx [file pone.0256457.s004.docx]

# We thank Dr Gao and one anonymous reviewer for their encouraging comments on our manuscript. Please, see our detailed response below.

# **Reviewer 1**

## **General comments:**

In the work “Colombia's bioregions as a source of useful plants”, the authors assembled a dataset of georeferenced collection localities of all vascular plants and useful plants of Colombia, and put all point locality information in a map of Colombia’s bioregions, and they found Andean, Amazon, Pacific, Llanos and Caribbean moist forests have the highest numbers of all vascular plant species and useful species descendingly. The manuscript is well written and data analysis is supportive of their conclusions. I think, with more work like this, may be conducted in other areas in the world, we will have a more specific layout of the useful plants and detailed conservation plans of the plant species in the Anthropocene during which nearly all the plant species face a high extinction risk. I am very happy to see such a work be published if they could address my following concerns:

1) It’s important to stress the importance of this or this kind of work. Collecting the information of useful plants in a specific area and showing the results publicly, I think, one of (those) aims to protect or conserve these valuable plant species, which should be addressed in the Introduction and Discussion. The authors may find it useful as a reference (Gao et al, Plant extinction excels plant speciation in the Anthropocene. *BMC Plant Biology* 2020, 20: 430. <https://doi.org/10.1186/s12870-020-02646-3>).

R: As suggested, we made some amendments to Introduction and Discussion, and we also added the following citation to the list of references:

Gao J-G, Liu H, Wang N, Yang J, Zhang X-L. Plant extinction excels plant speciation in the Anthropocene. BMC Plant Biol. 2020;20: 430. doi:10.1186/s12870-020-02646-3

2) The authors said “traditional knowledge and the importance of medicinal plants in Colombia that dates back to the last 30 years” and “Colombia ethnobotanical studies on indigenous populations began in 1860 with the work of Florentino Vezga”, which probably not the truth. As I knew, Alexander von Humboldt has traveled to South America (or Colombia) and collected many useful plants and carried them to Europe which was definitely before 1860 (which highlighted in their Acknowledgements). The authors may find it useful to read Wulf’s book (The invention of nature: The adventures of Alexander von Humboldt, the lost hero of science (John Murray, London, UK, 2015)).

R: Thank you for the suggestion, we now have updated these sentences with Humboldt but also with other references from the sixteen and eighteen centuries.

3) The authors explained the biodiversity of Pacific moist forests that “might have developed only in the last 5 million years once the landscape had changed into fully terrestrial”, which is very interesting. Can you expand it? (Including other bioregions of their manuscript) Linking plant evolutionary history and plant use is very crucial for our understanding of the current use scenarios of many plants. The authors may find it useful to refer Molina-Venegas’s recent work (Maximum levels of global phylogenetic diversity efficiently capture plant services for humankind. <https://doi.org/10.1038/s41559-021-01414-2>).

R: We have now added information about the origin of the flora for all bioregions except for the Caribbean one for which this information was more difficult to obtain. However, we did not include a specific link with useful plants because we don’t have enough evidence for doing this in the region and we feel this is out of the scope of the paper.

4) The authors indeed discussed their survey completeness and survey coverage of the useful plants, but not highlighted in the Discussion fully. The authors may find it useful to refer to a similar work “Ethnobotanical Knowledge Is Vastly Under-Documented in Northwestern South America” by Camara-Leret et al. (doi: 10.1371/journal.pone.0085794).

R: The above article was cited in the earlier draft of the manuscript (number 7 on the list of references). We agree that our study has some similarities with the work of Camara-Laret et al., however, the methods and materials were very different, which makes a comparison of the studies rather difficult. In particular, our study used the records of botanical collections, which were not necessarily assembled with plant uses in mind. In fact, some botanists who gathered data on distribution of Colombia’s useful plants might not even be aware of their useful attributes. We therefore limit our discussion of survey completeness and coverage by the reference to the results based on the analysis of species distribution datasets (such as GBIF), without making direct comparisons with the results of ethnobotanical studies, which we feel are out of the scope of the present work.

5) I strongly suggested moving Table S2.2 (Categories of plant uses following Diazgranados et al. (2020)) and Table S2.3 (Useful plants and their uses) to the main text. Because they are very crucial to understand some definitions used in this study. And one thing you should keep in mind, some plants are used both as Medicines and Human Food, or tri-usefulness (three or even more uses), which may be explained in the table caption.

R: As suggested, we moved Tables S2.2. and S2.3. to the main document (now Tables 1 and 2). We also edited the Table 2 caption as suggested.

6) In the discussion, they highlighted the importance of useful plants in promoting a bio-economy (reference 36 they cited), but the readers may be interested in the applications of these useful plants in achieving UN Sustainable Development Goals, especially in the face of COVID-19. For me, the useful plants in Colombia are key, at least, to some extent, in achieving Sustainable Development Goals 1 (No Poverty), 12 (Responsible Consumption and Production), 13 (Climate Action), and 15 (Life on Land). Can you expand it?

R: We already mentioned how medicinal plants have been highlighted under the current circumstances of the pandemic (l. 406-411). As suggested, we have added a small paragraph about the UN Sustainable Development goals (l.419-424).

## **Minor comments:**

Line 29: Formatting issue: gaps-,.

R: Corrected.

Line 203: Use abbreviations because you have already defined them (Line 157: annual mean temperature (ANMT) and total annual precipitation (ANP)).

R: Corrected.

Line 320: According to what? I believe you gave such an exact number (70%) must have based on some empirical study.

R: A reference to table S2.3 added.

Line 436: 3870/4200 is a high proportion, not “only”.

R: We agree with the comment; “only” has been deleted.

# **Reviewer 2**

There are some well-known problems with the used data, e.g. with GBIF, but the analyses seem to be sound and these data problems should not affect the general results of the study. So, I don't see any major technical issues.

R: We appreciate your positive comments. Yes, we recognized issues when using GBIF, but were extremely careful cleaning and checking the records we used for the analyses. With this revised version, we hope the manuscript is better now.
