# Supplementary material for: Colombia’s bioregions as a source of useful plants
Source: PLoS One. 2021 Aug 27;16(8):e0256457. doi: 10.1371/journal.pone.0256457 (PMC8396733; doi:10.1371/journal.pone.0256457)
Supplement: Attachment — Submitted filename: Response to Reviewers.docx [file pone.0256457.s005.docx]

# We thank an anonymous reviewer for their encouraging comments on our manuscript. Please, see our detailed response below.

Reviewer #1: (No Response)

Reviewer #3: I've been brought in to review this paper after one round of review has already been completed. I first read through the paper and then the response to reviewers letter, so that I could first make an opinion on the paper and then see if they have addressed previous criticism adequately.

Really I think this is an excellent paper, one which could provide a methodological template for future studies. The authors have been extremely thorough, not only in assembling and cleaning the data but also in analysing it. I only have minor suggestions. Had I been a first round reviewer, I might have made a couple of suggestions for alternative analyses that might provide some more perspectives on the sampling heterogeneity that affects the dataset. However the analyses shown in the paper are definitely sufficient, and I don't think it fair for a new reviewer to be brought in on the second round of reviews and start demanding new analyses; they would certainly not be essential to the paper which stands very well on its own merit. I will therefore only suggest that it might be worth showing, maybe as a supplementary figure, a scatterplot directly comparing within each bioregion, number of species to number of sampled/well sampled grid cells. Given the number of bioregions a correlation test would probably not be much use, but such a figure would provide quite a clear illustration a) of how strong the relationship is (and therefore potentially how much of the data signal is sampling), and which bioregions have substantially more/less data than would be expected given the extent of sampling. However I do recognise that the authors have already done substantial work on this paper and have definitely done enough to show their conclusions are valid, and so I won't insist on this and I think the paper is perfectly acceptable as is, barring a couple of wording and grammar quibbles:

R: As suggested by Reviewer, we added a supplementary figure (Fig. S2.6) comparing all vascular plant and useful plant species numbers to numbers of sampled grid cells within each bioregion. The same test for well sampled grid cells would require a much more complicated and time consuming data analysis. As the latter was not planned at the initial stages of the manuscript preparation, an additional analysis would mean substantial changes to Materials&Methods and Results sections, while being not essential to the paper as a whole. When a new figure S2.6 is considered, Amazon moist forests and Llanos savannas appear to be particularly data deficient relative to other regions; thus the scatterplots did not reveal any strong outliers in addition to those already discussed in the text of the manuscript. Therefore we did not make any changes to Discussion apart from making a brief reference to the figure.

Abstract line 29: “…contained the largest numbers of useful plants.” Phrasing is ambiguous; number of plants could be taken to mean absolute abundances. Maybe specify you mean largest number of useful plant *species*.

R: Changed as suggested.

Discussion, line 290: “…with 22.7% of well surveyed cells savanna had the lowest survey completeness.” Need comma after ‘cells’

R: Corrected.
